# Motivation levels and white matter microstructure in children living with HIV

**DOI:** 10.1038/s41598-024-54411-3

**Published:** 2024-02-23

**Authors:** Catherine J. Wedderburn, Tatum Sevenoaks, Jean-Paul Fouche, Nicole J. Phillips, Stephen D. Lawn, Dan J. Stein, Jacqueline Hoare

**Affiliations:** 1 Department of Paediatrics and Child Health, University of Cape Town, Cape Town, South Africa; 2 Neuroscience Institute, University of Cape Town, Cape Town, South Africa; 3 Department of Clinical Research, London School of Hygiene and Tropical Medicine, London, UK; 4 Department of Psychiatry and Mental Health, University of Cape Town, Cape Town, South Africa; 5 SA MRC Unit on Risk and Resilience in Mental Disorders, University of Cape Town, Cape Town, South Africa

**Keywords:** Motivation, Apathy, HIV, Children, Diffusion tensor imaging, HIV infections, Neuroscience, Brain, Public health, Human behaviour

## Abstract

Central nervous system involvement in HIV infection leads to neurobehavioural sequelae. Although apathy is a well-recognised symptom in adults living with HIV linked to alterations in brain structure, there is scarce research examining motivation in children living with HIV (CLWH). We used the Children’s Motivation Scale (CMS; normative mean = 50, SD = 10) to assess motivation levels in 76 CLWH aged 6–16 years (63 on antiretroviral therapy [ART]; 13 ART-naïve slow progressors) in South Africa. Overall, CLWH scored low on the CMS (mean = 35.70 [SD = 5.87]). Motivation levels were significantly reduced in children taking ART compared to ART-naïve slow progressors (p = 0.02), but were not correlated with markers of HIV disease (CD4 + cell count or viral load), or neurocognitive function (p > 0.05). CMS scores were correlated with diffusion tensor imaging metrics of white matter microstructure in specific frontostriatal brain regions (p < 0.05). On multiple regression, associations with the anterior limb of the internal capsule, a subcortical white matter region, remained significant after adjusting for potential confounders. These findings suggest that reduced motivation may be an important neurobehavioural symptom in CLWH and may reflect changes in white matter microstructure of frontostriatal brain regions.

## Introduction

There are 1.5 million children living with HIV (CLWH) globally^1^. HIV typically progresses quickly in children, and without antiretroviral therapy (ART) approximately half will die by the age of two years^2^, while many others suffer neurodevelopmental delay^3,4^. Involvement of the central nervous system (CNS) can lead to neurological, cognitive, psychological, and behavioural sequalae in CLWH^3,5–11^. The early initiation of ART has substantially improved child outcomes and shifted the focus to long-term neurobehavioural and academic outcomes, and daily functioning^12,13^, ensuring that children not only survive but also thrive. While in prior guidelines, ART initiation was informed by clinical status and CD4 levels, current guidelines recommend initiating ART on HIV diagnosis. However, only 54% of CLWH are estimated to be receiving ART with little improvement in recent years^14^. A minority of CLWH who receive no or minimal ART before the age of 10 years are able to maintain virological suppression while remaining clinically asymptomatic, also known as “ART-naïve slow progressors”^15,16^. Less is known about the outcomes of this population^17,18^. Understanding the neurobehavioural repercussions of HIV infection in children across the spectrum of disease is critical to optimise provision of care.

Neuropsychological impairment is estimated to occur in over 50% of CLWH without treatment^3,11^, and CLWH remain at-risk for deficits despite ART^5,19^. While various behaviours have been examined, to our knowledge only one study has specifically examined motivation levels in CLWH^20^. Motivation is an important psychological construct that describes the “*impetus that gives purpose or direction to behaviour in humans*”^21^. The lack of motivation has been documented as a negative symptom in many neuropsychiatric disorders^22^, and the impact of diminished motivation has been described in terms of daily living, relationships and employment as well as affecting disease outcomes and chronic medication adherence^23^. In the single report on motivation in CLWH, HIV was associated with reduced motivation^20^. In contrast, in adults living with HIV, lack of motivation is a well-recognised neuropsychiatric symptom^9,24–27^. In this literature, low motivation not caused by intellectual deficit, emotional disturbance or reduced consciousness, that leads to diminished goal-orientated emotional, cognitive and motor behaviour, is known as ‘apathy’^28,29^. Apathy has been shown to be present in the absence of depression in adults living with HIV^25,30–33^, and to impact daily function^34,35^, medication adherence^25,36^, and caregiver distress^37^. Conceptually, apathy in adults may not be the same as lack of motivation in children, therefore more research is needed to understand this concept in children. This is particularly important given motivation may substantially impact school outcomes, along with self-care and ART adherence^38,39^, which is often sub-optimal in children^40,41^. More research into motivation is needed to inform potential intervention strategies.

Studies examining the aetiology of lack of motivation in children of all ages remain scarce. Neuroimaging is a useful tool to understand neuroanatomical pathways underlying cognition and behaviour. Brain changes on neuroimaging have been described in CLWH compared to children without HIV with varying reports at different ages including frontal cortex and subcortical differences; white matter abnormalities are most frequently reported^42–44^. Diffusion tensor imaging (DTI) is a powerful technique for investigating white matter abnormalities seen in HIV^17,45,46^. White matter microstructural differences across tracts have been seen in CLWH compared to those without HIV^47–50^. Separately, some studies have found that DTI metric differences between children with and without HIV are associated with cognition^51,52^. Given frontostriatal circuits are known to play a role in motivation as well as cognition and behaviour^53–55^, and HIV is known to have a predilection for frontal and subcortical regions^56–58^, it may therefore be hypothesised that HIV-CNS disease in these regions of the brain may precipitate low motivation^24,59^. However, few studies have been published examining motivation and HIV-related structural brain deficits in children or adolescents. One study in adults showed an inverse correlation between the volume of the nucleus accumbens and apathy^59^. Two other adult studies have used DTI; one found an association with apathy and white matter tract change in prefrontal areas^33^, while another showed an association with white matter changes in frontal and basal ganglia regions^60^, Finally, one adolescent study showed association with impaired global white matter microstructure^61^. Involvement of the developing CNS makes CLWH particularly vulnerable to disease processes compared to adults^61^. Understanding the impact of HIV on neurostructural pathways involved in motivation, and the effect of ART, may help determine intervention priorities to support CLWH.

Separately, apathy has also been linked with neurocognitive impairment in some adult studies^24,34,62^, including deficits on dual-task performance^62^, assessments of cognition, psychomotor speed and verbal fluency^34^, and verbal learning and mental flexibility tasks^24^. However, others show contrasting results and found no evidence of an association between apathy and neurocognitive impairment^25,30^.

Given the lack of literature on the symptomatology, time course, neuropsychology, or neuroimaging associations of motivation in CLWH, the aim of this study was to examine the association between motivation and: (1) HIV disease markers (as assessed by CD4 + cell count, HIV viral load and treatment status); (2) cognitive function (as assessed by a validated neuropsychological test battery); and (3) white matter microstructure (using diffusion tensor imaging) in CLWH in South Africa. Our hypothesis-based focus was on frontostriatal regions due to prior research on motivation and the predilection of HIV for these areas.

## Methods

### Participants

This study was nested within a larger project for which ART-naïve and ART-treated CLWH were recruited from Infectious Diseases clinics in two Community Health Centres, and the paediatric neurology service of a tertiary children’s hospital in South Africa, to examine neurocognitive outcomes^17,52^. Inclusion and exclusion criteria are detailed in full elsewhere^17,52,63^. In summary, children aged 6–16 years diagnosed with perinatal HIV infection attending an out-patient clinic were included. Children on ART were eligible if they were stable on antiretrovirals for a minimum of 6 months, while children living with HIV who were asymptomatic slow progressors and ART-naïve were also included. Children who were ART-naive but not slow progressors were not included in this study. Those with a history of the following were excluded: medical comorbidities (unstable medical condition including epilepsy and diabetes mellitus); documented CNS neurological conditions (other than HIV); active tuberculosis; head injury with hospital admission or skull fracture; history of traumatic or neurodevelopmental disorder not attributed to HIV; significant perinatal conditions (prematurity, hypoxic ischaemic encephalopathy, jaundice requiring transfusion); maternal drug or alcohol use during pregnancy; clinically significant attention-deficit/hyperactivity disorder, depression, or anxiety that may have affected a child’s performance, assessed using the Beck Youth Inventory and the Conners Parents Rating Scale^63^; or MRI contraindications. A total of 76 perinatally HIV-infected children completed the Children’s Motivation Scale questionnaire and a comprehensive neuropsychological test battery and had MRI scans performed as part of the larger study procedures.

### Instruments and measures

Demographic and socioeconomic information was collected using a demographic questionnaire specifically designed for the purposes of this study as well as the Family Resources Scale (FRS). This is a 30-item scale developed to measure the sufficiency of various resources in homes with young children, including access to healthcare, income, and education. The scale is completed by a parent/caregiver and has been shown to have good test–retest and internal reliability and concurrent validity^64^.

#### Children’s Motivation Scale (CMS)

Motivation was measured using the Children’s Motivation Scale (CMS). The CMS is a validated 16-item parent-completed questionnaire developed to assess a child’s motivation^65^. The scale was developed from the Adult Evaluation Scale (AES) which has been used with success in adults living with HIV^29^. Parents rate their child’s level of motivation for various activities using a five-point Likert-type scale by responding to questions, for example asking if the child starts playing (games, activities) on his/her own or is interested in learning new things (such as the alphabet or a new sport). The CMS is available in the original publication^65^. It is scored by deriving a total score of the 16 items which may range from 0 to 64, where higher scores represent greater motivation and lower scores indicate poor motivation. Normative values have been reported as mean of 50, standard deviation of 10, with clinical values indicative of poor motivation on a US inpatient psychiatric unit and outpatient affective disorder clinic as mean of 31, standard deviation of 10^65^. This measure was forward and back translated and administered to participants in their home language by trained research staff.

#### Clinical measures

Treatment status of children was documented as either on ART or ART-naïve as per the parent study^17,66^. Current CD4 + cell count and HIV viral load data were collected from results of the most recent blood tests taken at outpatient clinics. Encephalopathy was assessed based on a comprehensive neuromedical assessment by a clinician specialised in paediatric neurodevelopment^67^.

#### Neuropsychological assessment

A neuropsychological test battery was administered to all children to assess global cognitive function and frontostriatal performance, as well as specific cognitive domains, of which attention, working memory, processing speed and executive function were examined in detail in this nested study. These domains have been specifically linked to apathy in adults and are sensitive to fronto-subcortical deficits associated with HIV. The test battery included the Weschler Intelligence Scale for Children (WISC-IV)^68^ Colour Trails 1^69^, Test of Everyday Attention for Children WISC Digit Span Backwards^68^, NEPSY-II Inhibition, Switching and Naming subtests^70^, Category and Phonetic fluency, Colour Trails 2^69^, and the Wechsler Abbreviated Scale of Intelligence Matrix Reasoning and Similarities subtests^68^. These are standardised neuropsychological assessments that are routinely used in paediatric clinical and research settings in South Africa and globally^17^. Test content and instructions were forward and back translated into the local language, isiXhosa, to ensure the original and translated versions matched each other, and were culturally appropriate^17^. All tests were administered to participants in their home language by trained research staff.

### Diffusion tensor imaging

#### Image acquisition

Diffusion Tensor Imaging (DTI) was performed at the Cape Universities Brain Imaging Centre (CUBIC), Tygerberg Hospital using a 3 T Siemens Allegra MRI scanner. Images were captured with a single-channel transmit-receive head coil in axial orientation with diffusion weighted parameters: TE = 88 ms, TR = 8800 ms, FOV = 22 cm, 65 slices, 1.8 × 1.8 × 2.0 mm^3^ spatial resolution, 0% distance factor and two-fold GRAPPA acceleration. Gradients were applied in 30 directions with b = 1000 mm/s^2^ and 3 directions with b = 0 mm/s^2^. The 5-min sequence was repeated 3 times per child, and acquisitions with poor image quality were excluded. All scans were clinically reported and children were referred on to the appropriate services if indicated.

#### Image processing

FSL (FMRIB Software Library) 4.8 toolbox was used to correct for eddy current and motion distortions^71^. Further preprocessing was then carried out in MATLAB (Mathworks, Natick, MA) R2012b using a series of in-house scripts. Images were affine registered to the first acquisition’s b = 0 mm/s^2^ image and outlier data points were excluded. Each child’s corrected acquisitions were then averaged and imported into FSL 4.8 for further processing. Fractional anisotropy (FA), mean diffusivity (MD), radial diffusivity (RD) and axial diffusivity (AD) image maps were generated using a linear fit of the tensor model to the diffusion-weighted data. Pre-processed data were visually checked to ensure that images presented with no significant distortions.

Following brain extraction with FSL BET^71^, with the fractional intensity threshold specified at 0.2 to exclude non-brain voxels, the tract-based spatial statistics process (TBSS) for whole-brain and region of interest (ROI) was used to analyse FA images^72^. As the adult template included with TBSS is not suitable for paediatric images, a single subject’s FA image was selected to be the study-specific target for registration. The target was identified by first registering every subject’s FA image to each other, and then choosing the image with the minimum mean displacement to be representative of the group.

All FA images were then aligned to the target image space and upsampled to MNI space, allowing for the previous estimated registration limits. A mean FA skeleton was then generated from the averaged thinned FA image. This skeleton corresponds to the centre of white matter tracts shared by the cohort and was thresholded at 0.2. All pre-aligned FA, MD, AD and RD data were projected onto the mean FA skeleton image, and a distance map created of each subject image from the skeleton. ROI masks were created to delineate white matter regions using the JHU-81 white matter atlas included with FSL^73^. Specific frontostriatal regions were selected a priori for inclusion based on literature assessing pathways for motivation as well as neuroimaging studies of HIV: corpus callosum genu, fornix, anterior limb of internal capsule, anterior and superior corona radiata, cingulum, and the superior longitudinal fasciculus. FA, MD, AD and RD values were extracted for these regions for analysis.

### Statistical analyses

Demographic and clinical variables, and motivation levels within the cohort were described. Data were assessed for normality and parametric or non-parametric tests were conducted accordingly. Independent t-tests, Chi-squared tests and ANOVAs were used to examine grouped data comparing sociodemographic variables between ART-naïve slow progressors and children on ART.

The primary outcome was child motivation as measured by the CMS. This was analysed as a continuous scale in order to maintain statistical power, however, different cut-offs were explored relating to previously published normative values^65^. Mean and standard deviation scores were calculated and compared with that of a normative population (mean 50, SD 10) and previously published values for a clinically apathetic population (mean of 31, SD 10).

CMS scores were compared between children who were treated with ART and ART-naïve slow progressors using unpaired t-tests. Correlation analyses were then conducted between CMS scores and the following variables: (i) child age and education; (ii) disease markers including current CD4 + cell count and viral load; and (iii) cognitive domain scores (derived by calculating an average of the included tests). Pearson’s or Spearman’s coefficients were used as appropriate based on data distribution.

Finally, we examined associations between white matter microstructure and motivation. Pearson’s correlations were performed between CMS score and FA, MD, RD and AD values in ROIs selected a priori. Any relationships that were significant at the bivariate level were further explored using multiple linear regression models including child age, sex and ART use as covariates.

All tests were 2-tailed with a significance level of p < 0.05. Analyses were completed using Stata version 14.2.

### Ethics

The Human Research Ethics Committee of the University of Cape Town’s Faculty of Health Sciences granted ethical approval for the larger research programme within which this one was nested. Written informed consent was obtained from parents/legal guardians for the child to participate in the study, and the child provided assent. The study was conducted in accordance with ethical standards and relevant institutional guidance.

## Results

### Demographic and clinical characteristics

A total of 76 CLWH who had a completed Children’s Motivation Scale (CMS) were included in this study. Demographic and clinical characteristics of all 76 eligible children are shown in Table 1. The median age was 9.9 years, ranging from 6 to 17 years, and 46% were female. A total of 59% children had repeated at least one grade at school and the median highest grade passed was 3, a reflection of years of completed schooling. The median family resources scale total was 71.5 out of a total 150 indicating a high perceived resource and support inadequacy. All children were perinatally infected with HIV. The median CD4 + cell count was 805 (standard deviation (SD) 713), 11% having a CD4 + cell count < 500, while 72.6% had undetectable viral loads. Half had a history of HIV-related illness including bronchitis, pneumonia, TB; no children reported current HIV-related illnesses.

**Table 1:** Demographic and clinical characteristics of children (n = 76).

Variables	Total N (%)	ART 63 (82.9%)	ART naive 13 (17.1%)	*p*
Sex (% female)	35 (46.1%)	27 (42.9%)	8 (61.5%)	0.22
Age (years): median (IQR)	9.9 (8.2–12.2)	10.1 (8.2–12.5)	9.7 (7.9–11.2)	0.30
Ethnicity				
Black African	71 (93.4%)	60 (95.3%)	11 (84.6%)	0.20
Mixed ancestry	5 (6.6%)	3 (4.8%)	2 (15.4%)	
Education				
Highest grade passed: median (IQR)	3.00 (1–4)	3 (1–5)	2.5 (1.5–3)	0.37
Repeated grades	44 (58.7%)	36 (57.1%)	8 (66.7%)	0.75
Primary caregiver age (years): median (IQR)	40.5 (36–49.5)	40 (36–51)	41 (34–47)	0.59
Caregiver marital status (married)	29 (38.2%)	24 (38.1%)	5 (38.5%)	0.98
Caregiver disability grant^#^	18 (24.3%)	16 (26.2%)	2 (15.4%)	0.50
Family resources scale total, median (IQR)	71.5 (63–85.5)	72 (54–87)	65 (55–82)	0.42
HIV related illness^†^	35 (50.0%)	34 (54.8%)	1 (12.5%)	0.06
CD4 + cell count, cells/mm^3^, median (IQR)	804.5 (562–1266)	901 (644–1333)	558 (515–684)	0.004*
< 500	8 (11.4%)	6 (10.3%)	2 (16.7%)	0.62
≥ 500	62 (88.6%)	52 (89.7%)	10 (83.3%)	
Viral load, copies/ml				
< 40 (undetectable)	45 (72.6%)	43 (79.6%)	2 (25.0%)	0.004*
40–10,000	7 (11.3%)	4 (7.4%)	3 (37.5%)	
> 10,000	10 (16.1%)	7 (13.0%)	3 (37.5%)	
Log HIV RNA viral load, median (IQR)	1.30 (1.30–1.60)	1.30 (1.30–1.30)	3.57 (1.45–4.43)	0.002*
ART use				
Naïve		13 (17.1)		
1st line		33 (43.4)		
2nd line		9 (11.8)		
3rd line		1 (1.3)		
On ART, no regimen given		20 (26.3)		

Median (interquartile range) as significant Shapiro–Wilk test, non-normal distribution.

^†^HIV-related illnesses: Nose bleeding (1), bronchitis (1), TB (24), Pneumonia (10), TB meningitis (1); Percentages calculated from available data.

^#^Disability grant: this is a national monetary grant provided to people with a physical or mental disability that means they are unfit to work for a period longer than six months. Missing data: Education variables n = 1; disability grant n = 2, HIV-related illness and CD4 + n = 6; viral load n = 14. Log10 viral load calculated by transforming undetectable values. Given the limit of detection is 40 copies/mL, we used a midway point of 20 copies/mL. *p < 0.05.

Of the included children, 13 (17%) children were ART-naïve while 63 (83%) were on ART. All children on ART had been on treatment for a minimum of 6 months; of those with regimen information available, 33/43 (76.7%) were still on triple first line ART, while 9 (20.9%) were on second line and 1 (2.3%) child was on third line. There were no significant between-group differences in demographic variables between ART-naïve (n = 13) and treated patients (n = 63) (Table 1). However, ART-treated patients had higher CD4 + cell counts (median 901 versus 558, p = 0.004), and lower log viral loads (median 1.30 versus 3.57, p = 0.002).

### Children’s motivation scale scores

CMS scores for the whole cohort were on a scale of 0 to 64 with lower scores reflecting more severe lack of motivation. The overall mean score on the CMS was 35.70 (SD 5.87) with a range from 22 to 52. The mean score was significantly lower than that of the normative population (mean of 50, SD 10) p < 0.0001. Further exploring the association between HIV and lack of motivation using the 25th percentile of the CMS (score of 32; equivalent to mean plus 2 SD below the CMS mean of averaged previously reported paediatric normative samples^65^) as a threshold indicated 28% of children in this group had substantial lack of motivation.

There were no significant correlations between motivation and age (rho = 0.03, p = 0.82) or education level (rho = 0.001, p = 0.99) (Supplementary Table S1), and no difference in CMS score by child sex (t-test = − 0.22, p = 0.83).

### Motivation and markers for disease progression

CMS scores were not correlated with either absolute CD4 + cell count (rho) = − 0.16, p = 0.19) or HIV viral load (rho = 0.06, p = 0.63) (Supplementary Table S1). Similarly, there were no differences comparing categorical CD4 + cell count < 500 or > 500 (p = 0.61), detectable versus undetectable viral load (p = 0.35), or children with and without a history of HIV-related illness (p = 0.87).

**Figure 1:**
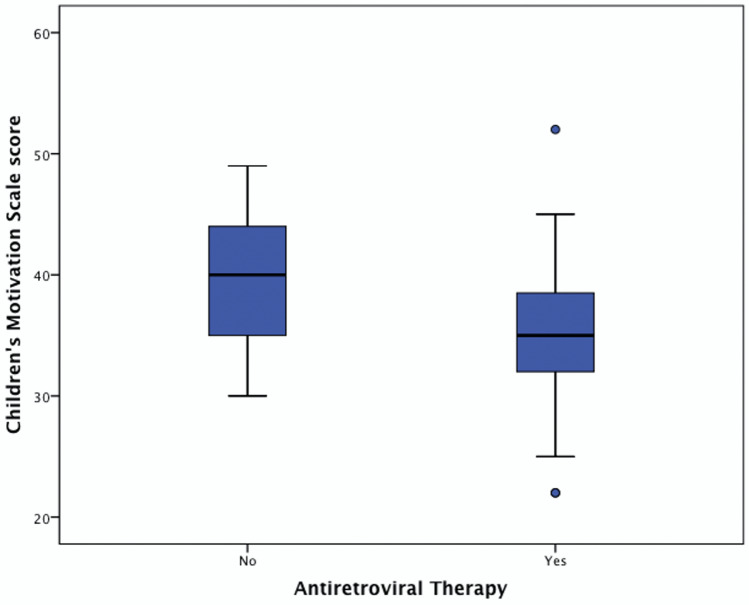
A comparison of child motivation scores between ART-treated and ART-naive children. Scores are presented as the Children’s Motivation Scale standard score. The box-and-whisker plots demonstrate the data distribution and group differences of the child motivation scores, p < 0.05.

A comparison of the scores of children based on ART showed the mean CMS score for ART-naïve slow-progressor children was 39.15 (6.12) versus ART-treated 34.98 (5.60), and the ART-treated children demonstrated significantly lower CMS scores (t: 2.40, p = 0.02) (Fig. 1). Given children on ART had lower scores, we went on to examine whether there was any effect of prior encephalopathy. 18 children had been given a diagnosis of HIV encephalopathy prior to entering the study and all were established on ART. A sub analysis of those children on ART with (n = 18) and without encephalopathy (n = 45) showed they did not significantly differ in CMS score (mean 34.56 (6.35) versus 35.16 (5.35), t = 0.38, p = 0.70).

### Motivation and cognition

Overall, all cognitive domain z-score means were below 0, indicating children scored below the equivalent average standard from HIV-negative data with z-scores ranging from − 0.16 to − 0.56. There were no significant correlations (p < 0.05) found between cognitive domain scores, global, or frontostriatal composite scores and the CMS (Table 2).

**Table 2:** Correlations between cognitive domains scores and child motivation scores.

Cognitive domain	Neuropsychological Tests	Mean (SD) of z-score	Correlation coefficient	p-value
Attention	Weschler Intelligence Scale for Children (WISC-IV), Colour Trails 1, Test of Everyday Attention for Children (TEA-Ch)	− 0.46 (0.60)	− 0.06	0.63
Executive function	NEPSY-II Inhibition, Switching and Naming subtests, Category and Phonemic Fluency, Colour Trails 2, Wechsler Abbreviated Scale of Intelligence Matrix Reasoning and Similarities subtests	− 0.33 (0.57)	0.15	0.19
Working memory	WISC Digit Span Backwards	− 0.16 (1.00)	− 0.20^**†**^	0.08
Processing speed	WISC-IV Processing Speed Index	− 0.56 (1.05)	0.01^**†**^	0.90
Frontostriatal composite	Combines test scores from attention, processing speed and executive functioning cognitive domains	− 0.39 (0.64)	0.12	0.28
Global cognitive composite	Combines scores from attention, processing speed, memory, executive function, general intellectual function, motor function, visual spatial ability and language	− 0.27 (0.42)	0.06	0.64

The tools were translated and back-translated and administered in the child’s language of preference. Age-adjusted z-scores were calculated using norms generated from local controls given that there are no normative data for South Africa; 0 signifies that the child is performing at an average standard equivalent to the control group, below 0 indicates performance below average. Pearson’s correlations were used unless ^†^ Shapiro–wilk test for normality significant p < 0.05 when Spearman’s rank correlation coefficients are presented. Results of correlations between individual neuropsychological tests and child motivation scores were also not significant.

### Motivation and Neuroimaging

Of the 66 children with DTI, 61 neuroimaging sequences were included in the final neuroimaging analysis and 5 children were excluded due to motion artefacts. There were no significant differences in underlying demographic and clinical variables (age, sex, race, education, CD4 + cell count, viral load, ART) between the 61 children included, and the 15 children without scan data (Supplementary Table S2). Mean FA values for regions of interest by ART group are included in Supplementary Table S3.

**Figure 2:**
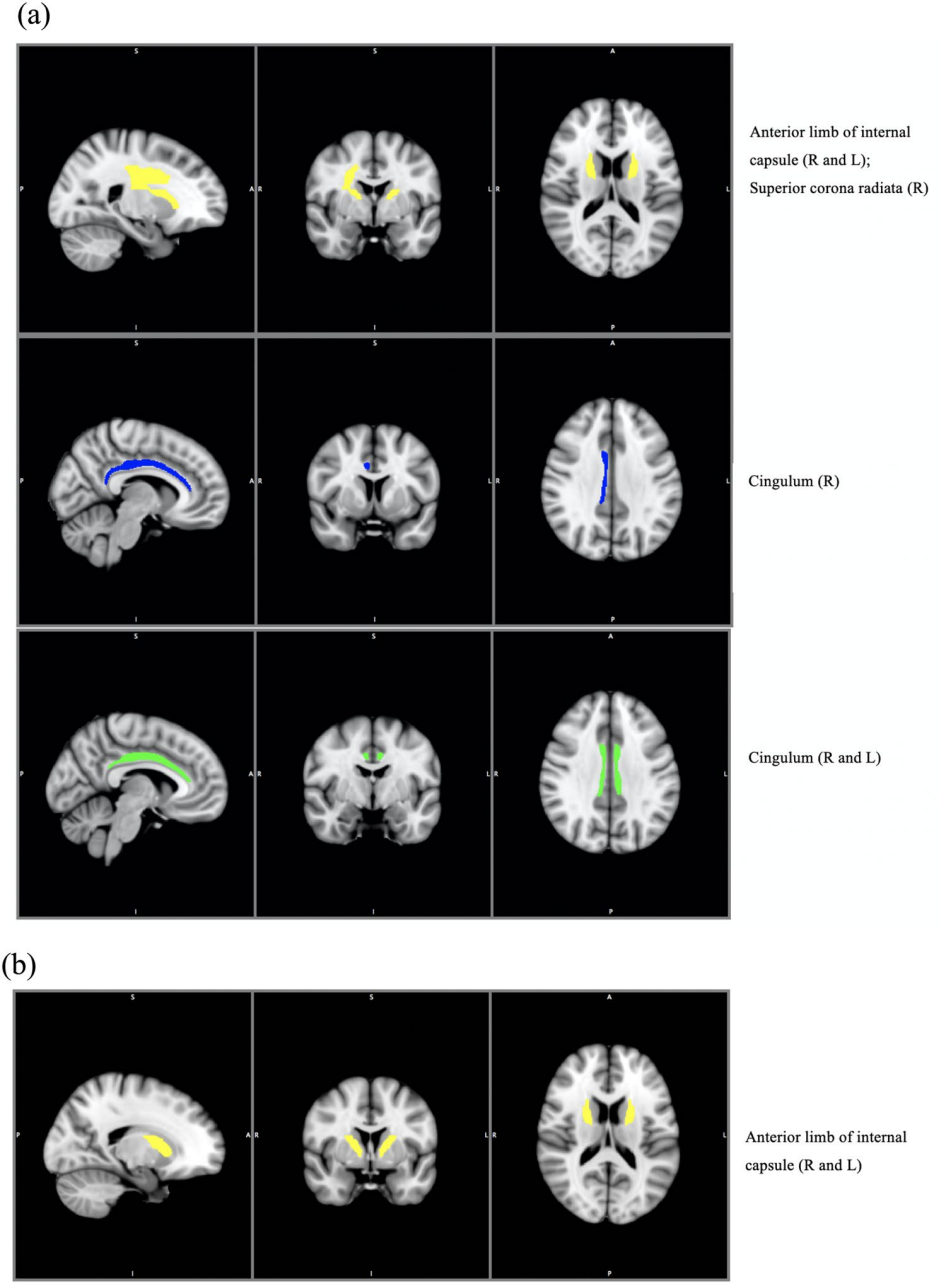
White matter tracts associated with child motivation score. (**a**) White matter tracts with significant correlations between child motivation score and the different DTI metrics. (**b**) White matter tracts that remained associated with child motivation score on multiple linear regression. White matter tracts superimposed on a 3D brain template. From left to right: Sagittal, coronal, and axial views. S: superior, I: Inferior, A: anterior, P: Posterior, R: Right, L: Left. Colour coded: Fractional Anisotropy: yellow; Mean Diffusivity: blue; Radial Diffusivity: green.

Bivariate analyses of CMS score and DTI metrics were undertaken within a priori regions. Statistically significant correlations between CMS and FA, MD and RD are shown in Table 3 and Fig. 2a (there were no significant correlations in AD). Negative correlations were observed between CMS and FA in the anterior limb of the internal capsule bilaterally and the superior corona radiata (right), and positive correlations with MD in the cingulum (right), and RD in the cingulum (bilaterally).

**Table 3:** Correlations between diffusion parameters and child motivation score by region-of-interest.

Brain region	Hemisphere	FA		MD		RD		AD	
		r	p	r	p	r	p	r	p
Corpus callosum genu		− 0.13	0.30	0.00	0.99	0.01	0.93	− 0.02	0.89
Fornix		− 0.17	0.20	0.09	0.47	0.15	0.25	− 0.06	0.64
Anterior limb of internal capsule	R	− 0.28	0.03*^†^	0.15	0.24	0.24	0.06	− 0.06	0.67
	L	− 0.30	0.02*^†^	0.15	0.27	0.25	0.05	− 0.07	0.59
Anterior corona radiata	R	− 0.22	0.09	0.07	0.62	0.11	0.39	− 0.02	0.86
	L	− 0.15	0.26	0.21	0.11	0.21	0.11	0.09	0.49
Superior corona radiata	R	− 0.26	0.04*	0.07	0.58	0.16	0.21	− 0.10	0.44
	L	− 0.16	0.22	0.23	0.08	0.24	0.07	0.03	0.83
Cingulum	R	− 0.22	0.09	0.31	0.02*	0.30	0.02*	0.15	0.24
	L	− 0.22	0.09	0.24	0.06	0.26	0.04*	0.10	0.46
Superior longitudinal fasciculus	R	− 0.02	0.86	− 0.22	0.10	− 0.20	0.12	− 0.23	0.07
	L	0.12	0.35	− 0.21	0.10	− 0.21	0.10	− 0.21	0.10
Superior fronto-occipital fasciculus	R	− 0.21	0.11	0.08	0.57	0.13	0.31	− 0.10	0.43
	L	− 0.14	0.29	− 0.04	0.79	0.02	0.90	− 0.10	0.45

Pearson’s r correlations (2-tailed) presented.

*FA* fractional anisotropy, *MD* mean diffusivity, *RD* radial diffusivity, *AD* axial diffusivity, *R* right, *L* left.

*p < 0.05 in bivariate analyses.

^†^Significant correlation at p < 0.05 after controlling for age, sex and ART use.

For ROIs which showed significant bivariate correlations, multiple regression analyses were performed with CMS score as the dependent variable, DTI values as predictors, and age, sex and ART use as covariates. FA values in the right and left anterior limb of the internal capsule remained significant predictors of CMS score (beta = − 0.32, p = 0.02, and beta = − 0.29, p = 0.04 respectively) (Table 3, Fig. 2b; Supplementary Table S4). ART use also accounted for some of the CMS score in these regions (beta = − 0.32, p = 0.01, and beta = − 0.28, p = 0.03).

## Discussion

This study investigated motivation levels, clinical and cognitive correlates, and white matter microstructure in CLWH. We found that: (1) CLWH scored low on the CMS indicating poor motivation levels; (2) CLWH treated with ART had significantly lower motivation levels compared to ART-naïve slow progressors; (3) there were no correlations between motivation levels and cognitive function or current markers of HIV disease; however, (4) there were significant associations between motivation levels and white matter microstructure in specific frontostriatal regions, particularly the anterior limb of the internal capsule.

Apathy has been well-described in the adult HIV literature and this study demonstrates that problems with motivation may also affect children. This may have key implications given ongoing problems described in school performance in CLWH^74^. Our results are consistent with the one previously published study examining motivation in CLWH, suggesting lack of motivation may be an important symptom in CLWH^20^. Developmental psychology describes how infants are born with an intrinsic motivation^75^, however, in our study most CLWH fell below the normal motivation range previously reported^65,76,77^, and over a quarter present with substantial lack of motivation. This prevalence is similar to estimations of clinically significant apathy (26%) in adults living with HIV^26^.

Lower motivation levels were found in the ART-treated group compared to ART-naïve slow progressor children. CLWH on ART are more likely to have had a faster disease progression leading to ART initiation given prior HIV guidelines where ART initiation was based on disease severity, compared to ART-naïve slow progressor children who have not had an AIDS-defining illness and are able to maintain virological suppression without treatment^18^. Thus, it may be inferred that although lack of motivation can occur prior to significant immune suppression, it has been shown here to occur more frequently in those treated with ART potentially related to more extensive HIV CNS invasion. This is particularly concerning as CLWH are often on complicated ART regimens with multiple side effects and need to be motivated to maintain optimum medication adherence^40,41^, and adult studies indicate an association between apathy and poorer medication adherence^36,78,79^.

Motivation levels did not correlate with age, education, or laboratory markers of HIV disease (current CD4 + cell count, viral load). These results are consistent with adult literature suggesting apathy may present without major immune compromise^26^, and may not show a relationship with current CD4^9,24,34^. The general level of neurocognitive function was low, similar to prior reports showing cognitive impairments in CLWH compared to controls^52^. However, there was no evidence of an association between motivation levels and cognitive scores in the executive function, attention, processing speed and working memory domains. This finding is similar to that of other research in adults using varying measures of apathy^25,30^, although the literature from adults is inconsistent as some studies show an association between apathy and cognition^9,59,80^. Previous studies have indicated that the relationship between cognition and apathy is mainly seen in adults with HIV who had greater cognitive impairment^24^, and it is possible the children in this study did not have the same degree of impairment to show an association. In addition, motivation and cognitive processes are in different circuits in the brain which may require substantial damage before both are affected^59^.

Using DTI, we identified a relationship between motivation score and white matter microstructure, similar to adult studies of apathy and HIV^33,60^. In particular, DTI metrics in the anterior limb of the internal capsule, superior corona radiata, and the cingulum were correlated with CMS score suggesting white matter integrity in these regions is associated with motivation. The anterior limb of the internal capsule is a white matter subcortical structure situated between the caudate nucleus (medially) and the lentiform nucleus (laterally). It predominantly contains thalamic and brainstem fibres connecting the thalamus with the prefrontal cortex (thalamocortical and corticothalamic) and the frontal lobe with the pontine nuclei (frontopontine), as well as transverse fibres connecting the caudate and putamen^81,82^. The anterior limb of the internal capsule is known to be important in frontostriatal pathways that are often affected by HIV^56,57,83^. Frontostriatal circuits connecting the frontal cortex and basal ganglia are known to be involved in motivation^53^, and damage to this area has been suggested as the common mechanism leading to apathy in HIV in adults^9,59^. Microstructural differences between CLWH and controls have been described irrespective of ART status in these regions^52,61^. Suggested mechanisms include dopamine deficits^84^, and direct effects of HIV-1 proteins leading to synaptic dysfunction and dysregulation of motivational processes^85,86^.

Higher motivation was associated with lower FA, a measure of white matter integrity, whilst lower motivation was associated with higher MD and RD^17,87,88^ in the specific frontostriatal regions. The direction of effect is similar to correlations between DTI metrics and motivation that have been previously reported in adolescents^89^, however, it differs to some previous research in adults living with HIV^33^. In this case, findings in children may be separate to adults, and we hypothesise that decreased FA may represent more axons with increased numbers of crossing fibres in the white matter matrix causing a paradoxical decrease in FA^46^. This fits with results from other HIV studies^66,90^, particularly those examining the internal capsule^91^.

Strengths of this study lie in a well-characterised sample of children, with validated neurocognitive tests, and unique neuroimaging data from South Africa, which has the highest number of CLWH worldwide. However, several limitations are to be noted, firstly the cross-sectional design of this study means it is not possible to infer causality. Longitudinal studies are needed to understand the trajectory of motivation in comparison to controls over time. Secondly, there was heterogeneity in the sample and current CD4 + cell count and viral load are time-dependent variables which may not be optimum markers of disease activity; CD4 nadir data were not available but this would be useful to examine in future studies, along with investigating the effects of specific antiretroviral drugs and greater child numbers to allow more detailed characterization. However, this is considered a large sample size in the child HIV neuroimaging literature. Third, there are no validated clinical cut-off values for apathy, and in the absence of normative data for South African paediatric populations the only comparison we can make is with the original population published in the literature; further validation in low and middle-income countries is needed, along with research into whether parents’ perception of child motivation may be biased by illness, and development of appropriate child-completed measures of motivation. Fourth, while diverse regions have been described to be affected by HIV across cross-sectional DTI studies at different ages, in this study we focused on frontostriatal regions for their association with motivation. Given the potential for a diffuse effect of HIV, future studies should aim to explore the involvement of other brain regions with large enough populations to withstand multiple comparisons. Lastly, we do not have an HIV negative control group. Motivation in children is complex and it may not be possible to isolate from other external and internal factors including the environment, parenting behaviour, or exposure to trauma, which play a key role in motivation levels in children^92^. Further research studies are warranted that compare children living with and without HIV to understand this construct further.

## Conclusions

In conclusion, the results suggest that lack of motivation may be an important neurobehavioural symptom in CLWH, reflecting changes in white matter microstructure in frontostriatal regions, and which has implications for clinical care. As ART usage continues to increase, the population of CLWH progressing into adolescence is growing and long-term neuropsychological sequelae are becoming increasingly important to understand. This study demonstrates the value of assessing motivation in CLWH, and further work is needed to assess the possible value of motivation as a treatment target.

### Supplementary Information

Supplementary Tables.

## Data Availability

The data presented in this study are available upon reasonable request from the corresponding author*.*
