# Supplementary material for: Motivation levels and white matter microstructure in children living with HIV
Source: Sci Rep. 2024 Feb 23;14:4425. doi: 10.1038/s41598-024-54411-3 (PMC10891087; doi:10.1038/s41598-024-54411-3)
Supplement: Supplementary file 1 — Supplementary Tables. [file 41598_2024_54411_MOESM1_ESM.pdf]

**Supplementary Information**

# Motivation levels and white matter microstructure in children living with HIV

Catherine J Wedderburn[1,2,3], Tatum Sevenoaks[4], Jean-Paul Fouche[4], Nicole J Phillips[4], Stephen D Lawn[3], Dan J Stein[2,4,5], Jacqueline Hoare[2,4]

**Affiliations**

[1] Department of Paediatrics and Child Health, University of Cape Town, South Africa

[2] Neuroscience Institute, University of Cape Town, South Africa

[3] Department of Clinical Research, London School of Hygiene & Tropical Medicine, UK

[4] Department of Psychiatry and Mental Health University of Cape Town, South Africa

[5] SA MRC Unit on Risk and Resilience in Mental Disorders, University of Cape Town, South Africa

**This pdf contains the following information:**

**Supplementary Table 1:** Correlation matrix of CMS scores, demographic and clinical characteristics

**Supplementary Table 2:** Demographic and clinical characteristics of children who did and did not have imaging

**Supplementary Table 3:** Mean factional anisotropy values for regions of interest by ART treatment group

**Supplementary Table 4:** Multiple Linear Regression models

**Supplementary Table 1: Correlation matrix of CMS scores, demographic and clinical characteristics**

| Variable | CMS | CD4 | VL | Age | Education |
|---|---|---|---|---|---|
| **CMS** | - | -0.16 | 0.06 | 0.03 | 0.001 |
| **CD4** | -0.16 | - | -0.50** | -0.36** | -0.28* |
| **VL** | 0.06 | -0.50** | - | 0.11 | 0.14 |
| **Age** | 0.03 | -0.36** | 0.11 | - | 0.88** |
| **Education** | 0.001 | -0.28* | 0.14 | 0.88** | - |

**Notes***:* All variables except CMS were non-normally distributed, therefore non-parametric Spearman's rank correlation coefficients are presented.
CMS: Children's Motivation Scale; Education: highest grade passed
*$p$ = 0.01 to p<0.05. **p<0.01

**Supplementary Table 2: Demographic and clinical characteristics of children who did and did not have imaging**

| *Demographic Characteristics* | *Imaging* (n=61) | *No imaging* (n=15) | *P* |
|---|---|---|---|
| **Female Gender,** n (%) | 27 (44.3) | 8 (53.3) | 0.53 |
| **Age: median (IQR)** | 10.1 (8.4 to 12.5) | 9.1 (7.8 to 11.2) | 0.164 |
| **Ethnicity** | | | |
| Black African | 57 | 14 | 1.00 |
| Mixed Ancestry | 4 | 1 | |
| **Education:** Highest grade passed, median (IQR) | 3 (2-4) | 2 (1-3) | 0.11 |
| **ART treatment**, n (%) | 51 | 12 | 0.71 |
| **CD4**, median (IQR) | 806 (544 to 1266) | 803 (592 to 1443) | 0.58 |
| **Viral load**, median (IQR) | 1.3 (1.3 to 1.6) | 1.3 (1.3 to 4.43) | 0.36 |

Tests: Chi-squared or Fisher's exact test for categorical variables; Mann Whitney U for continuous variables. Missing variables: CD4 (n=6), viral load (n=14)

**Supplementary Table 3: Mean factional anisotropy values for regions of interest by ART treatment group**

| Brain region | Hemisphere | Fractional Anisotropy | |
|---|---|---|---|
| | | ART<br>Mean (SD) | ART naïve<br>Mean (SD) |
| Corpus Callosum genu | | 0.310 (0.032) | 0.308 (0.020) |
| Fornix | | 0.461 (0.040) | 0.458 (0.021) |
| Anterior limb of internal capsule | R | 0.407 (0.033) | 0.407 (0.022) |
| | L | 0.401 (0.031) | 0.393 (0.019) |
| Anterior corona radiata | R | 0.468 (0.039) | 0.456 (0.030) |
| | L | 0.376 (0.037) | 0.365 (0.025) |
| Superior corona radiata | R | 0.359 (0.038) | 0.349 (0.020) |
| | L | 0.306 (0.029) | 0.307 (0.017) |
| Cingulum | R | 0.347 (0.034) | 0.344 (0.010) |
| | L | 0.360 (0.035) | 0.352 (0.012) |
| Superior longitudinal fasciculus | R | 0.436 (0.047) | 0.402 (0.028) |
| | L | 0.290 (0.058) | 0.258 (0.071) |
| Superior fronto-occipital fasciculus | R | 0.425 (0.045) | 0.411 (0.034) |
| | L | 0.465 (0.034) | 0.458 (0.028) |

**Supplementary Table 4: Multiple Linear Regression models**

| Variable | B | Beta | T | P | 95% CI for B: lower | 95% CI for B: upper |
|---|---|---|---|---|---|---|
| **Model summary:** $R^2$= 0.18, F(4, 56)=3.01, p = 0.03 | | | | | | |
| **FA anterior limb of internal capsule, RH** | -61.27 | -0.32 | -2.32 | 0.02* | -114.30 | -8.24 |
| **Covariates** | | | | | | |
| Age | 0.15 | 0.06 | 0.44 | 0.66 | -0.53 | 0.83 |
| Gender | -0.48 | -0.04 | -0.32 | 0.75 | -3.54 | 2.57 |
| ART use | -5.12 | -0.32 | -2.57 | 0.01* | -9.10 | -1.13 |
| **Model summary**: $R^2$= 0.16, F(4,56)=2.77, p = 0.04 | | | | | | |
| **FA: anterior limb of internal capsule, LH** | -58.05 | -0.29 | -2.12 | 0.04* | -112.85 | -3.25 |
| **Covariates** | | | | | | |
| Age | 0.08 | 0.03 | 0.23 | 0.82 | -0.59 | 0.74 |
| Gender | -0.34 | -0.03 | -0.22 | 0.83 | -3.41 | 2.73 |
| ART use | -4.55 | -0.28 | -2.27 | 0.03* | -8.57 | -0.54 |
| **Model summary:** $R^2$= 0.14 , F(4,56)= 2.33, p = 0.07 | | | | | | |
| **FA: superior corona radiata, RH** | -37.76 | -.22 | -1.71 | 0.09 | -82.09 | 6.57 |
| **Covariates** | | | | | | |
| Age | -0.04 | -0.02 | -0.11 | 0.91 | -0.69 | 0.62 |
| Gender | -0.14 | -0.01 | -0.09 | 0.93 | -3.23 | 2.96 |
| ART use | -4.52 | -0.28 | -2.22 | 0.03* | -8.60 | -0.44 |
| **Model summary:** $R^2$= 0.14, F(4,56)= 2.31, p = 0.07 | | | | | | |
| **MD: cingulum, RH** | 54822 | 0.25 | 1.68 | 0.10 | -10289 | 119933 |
| **Covariates** | | | | | | |
| Age | 0.11 | 0.04 | 0.29 | 0.77 | -0.61 | 0.82 |
| Gender | 0.19 | 0.02 | 0.12 | 0.90 | -2.91 | 3.29 |
| ART use | -3.58 | -0.22 | -1.66 | 0.10 | -7.91 | 0.75 |
| **Model summary:** $R^2$= 0.15, F(4,56)=2.52, p = 0.05 | | | | | | |
| **RD: cingulum, RH** | 50756 | 0.29 | 1.90 | 0.06 | -2699 | 104210 |
| **Covariates** | | | | | | |
| Age | 0.18 | 0.07 | 0.48 | 0.63 | -0.56 | 0.91 |
| Gender | -0.03 | -0.00 | -0.02 | 0.98 | -3.10 | 3.04 |
| ART use | -3.94 | -0.24 | -1.91 | 0.06 | -8.09 | 0.20 |
| **Model summary:** $R^2$= 0.13 , F(4,56) = 2.12, p = 0.09 | | | | | | |
| **RD: cingulum, LH** | 39604 | 0.24 | 1.48 | 0.15 | -14146 | 93355 |
| **Covariates** | | | | | | |
| Age | 0.15 | 0.06 | 0.38 | 0.71 | -0.63 | 0.93 |
| Gender | -0.21 | -0.02 | -0.13 | 0.90 | -3.33 | 2.92 |
| ART use | -4.14 | -0.26 | -1.98 | 0.05 | -8.34 | 0.05 |

**Notes:** CMS: Dependent variable; *p<0.05
